# MITE insertion-dependent expression of CitRKD1 with a RWP-RK domain regulates somatic embryogenesis in citrus nucellar tissues

**DOI:** 10.1186/s12870-018-1369-3

**Published:** 2018-08-13

**Authors:** Takehiko Shimada, Tomoko Endo, Hiroshi Fujii, Michiharu Nakano, Aiko Sugiyama, Genya Daido, Satoshi Ohta, Terutaka Yoshioka, Mitsuo Omura

**Affiliations:** 1 National Agriculture and Food Research Organization, Institute of Fruit and Tea Tree Science, Shimizu, Shizuoka, 424-0292 Japan; 2 Faculty of Agriculture, Shizuoka University, Suruga, Shizuoka, 422-8529 Japan

**Keywords:** Somatic embryogenesis, RKD, Citrus, DNA marker, Egg cell, Apomixis, *CiFT* co-expression system

## Abstract

**Background:**

Somatic embryogenesis in nucellar tissues is widely recognized to induce polyembryony in major citrus varieties such as sweet oranges, satsuma mandarins and lemons. This capability for apomixis is attractive in agricultural production systems using hybrid seeds, and many studies have been performed to elucidate the molecular mechanisms of various types of apomixis. To identify the gene responsible for somatic embryogenesis in citrus, a custom oligo-DNA microarray including predicted genes in the citrus polyembryonic locus was used to compare the expression profiles in reproductive tissues between monoembryonic and polyembryonic varieties. The full length of *CitRKD1*, which was identified as a candidate gene responsible for citrus somatic embryogenesis, was isolated from satsuma mandarin and its molecular function was investigated using transgenic ‘Hamlin’ sweet orange by antisense-overexpression.

**Results:**

The candidate gene *CitRKD1*, predominantly transcribed in reproductive tissues of polyembryonic varieties, is a member of the plant RWP-RK domain-containing protein. *CitRKD1* of satsuma mandarin comprised two alleles (*CitRKD1-mg1* and *CitRKD1-mg2*) at the polyembryonic locus controlling embryonic type (mono/polyembryony) that were structurally divided into two types with or without a miniature inverted-repeat transposable element (MITE)-like insertion in the upstream region. *CitRKD1-mg2* with the MITE insertion was the predominant transcript in flowers and young fruits where somatic embryogenesis of nucellar cells occurred. Loss of *CitRKD1* function by antisense-overexpression abolished somatic embryogenesis in transgenic sweet orange and the transgenic T_1_ plants were confirmed to derive from zygotic embryos produced by self-pollination by DNA diagnosis. Genotyping PCR analysis of 95 citrus traditional and breeding varieties revealed that the *CitRKD1* allele with the MITE insertion (polyembryonic allele) was dominant and major citrus varieties with the polyembryonic allele produced polyembryonic seeds.

**Conclusion:**

*CitRKD1* at the polyembryonic locus plays a principal role in regulating citrus somatic embryogenesis. *CitRKD1* comprised multiple alleles that were divided into two types, polyembryonic alleles with a MITE insertion in the upstream region and monoembryonic alleles without it. *CitRKD1* was transcribed in reproductive tissues of polyembryonic varieties with the polyembryonic allele. The MITE insertion in the upstream region of *CitRKD1* might be involved in regulating the transcription of *CitRKD1*.

**Electronic supplementary material:**

The online version of this article (10.1186/s12870-018-1369-3) contains supplementary material, which is available to authorized users.

## Background

Somatic embryogenesis in nucellar tissues of citrus species is an apomictic system and genetically uniform clones with the same genotype as the maternal plant can feasibly be produced by sowing the seeds despite their highly heterozygous genomes [16]. This capability for apomixis is attractive in agricultural production systems using hybrid seeds, and many researchers have investigated the molecular mechanisms of various types of apomixis [20]. Among the various types of apomixis, citrus apomixis, in which a somatic embryo develops in nucellar tissues, is classified as sporophytic apomixis [37]. Major citrus varieties, such as satsuma mandarin (*Citrus unshiu* Marc.), sweet orange (*C. sinensis* (L.) Osbeck), grapefruit (*C. paradisi* Macfad.), and ponkan mandarin (*C. reticulata* Blanco), generally develop one or more somatic embryos that are genetically identical to the mother tree in addition to or instead of a zygotic embryo in the seed. This ability to generate multiple somatic embryos and a zygotic embryo in the same ovary tissue is called polyembryony in citrus. In citrus breeding, polyembryony frequently hampers the efforts to obtain zygotic embryos from sexual crosses because somatic embryos grow preferentially to zygotic embryos. Therefore, monoembryonic varieties are generally selected as the seed parent in cross breeding, which is a limitation to breeding because it reduces the available mating combinations. While, polyembryony is useful in rootstock propagation and genetically uniform rootstocks can feasibly be prepared solely by sowing seeds despite the highly heterozygous genomes of citrus species.

To date, various studies have been conducted to investigate the molecular mechanism of citrus adventive embryogenesis, as well as those of other types of apomixis [20]. Polyembryony is dominantly inherited into offspring according to observations of segregation in various cross populations [15]. It is conceivable that a single or a few genes are involved in the somatic embryogenesis and several molecular markers linked to a polyembryonic locus controlling embryonic type (mono/polyembryony) have been developed [11, 17, 29]. In our previous studies [29, 30], a major polyembryonic locus was located on linkage group 1 of the mandarin standard genetic map (AGI map) [36] and scaffold 1 of the clementine mandarin (*C. clementina* hort ex. Tanaka) genome sequence [42]. Subsequently, molecular tagging of the polyembryonic locus and construction of haplotype-specific bacterial artificial chromosome (BAC) contigs for the polyembryonic locus were carried out. Thereafter, the genomic region of the polyembryonic locus spanning approximately 380 kbp was sequenced and 70 open reading frames (ORFs) were predicted from genomic sequences [28]. Transcription-based approaches have been used to explore the genes associated with citrus somatic embryogenesis. Various genes with specific transcription profiles in either monoembryonic or polyembryonic varieties have been identified by subtractive suppression hybridization (SSH) and microarray analyses [10, 22, 27]. In these studies, heat shock-related proteins (HSPs) were predominantly expressed among polyembryonic variety genes, as well as WRKY, WD40, and serine carboxypeptidase (SCP) genes.

Recently, next-generation sequence (NGS) technology has allowed rapid and comprehensive sequencing analyses for whole genomes and transcripts of target tissues and organisms. Using NGS technology, the regulatory genes involved in somatic embryogenesis were explored and it was proposed that miRN23-5p-Cs9g06920, a micro-RNA (miRNA, a type of non-coding RNA with a typical length of 20–24 nucleotides), likely has a major role in regulating somatic embryogenesis [23]. It was reported that *CitRWP* encoding a RWP-RK domain-containing protein [35] would regulate somatic embryogenesis because the insertion of a miniature inverted-repeat transposable element (MITE) was found only in the *CitRWP* genes of polyembryonic citrus varieties in NGS-based comparative genomic sequence analysis and the transcription level of *CitRWP* in their ovules was higher than in monoembryonic varieties [41]. The MITE co-segregated with poly/monoembryonic type in a segregation population of 217 seedlings. In *Arabidopsis*, RKD genes containing the RWP-RK domain have been characterized as important regulators of an egg cell-related gene expression program [35]. However, functional validation of these candidate genes through transcriptome and NGS analyses remains to be done.

Here, to identify the candidate RKD gene (*CitRKD1*) responsible for somatic embryogenesis in citrus, a custom oligo-DNA microarray was designed using ORFs newly predicted from a 380 kbp draft sequence of the polyembryonic locus [28], and was used to compare the transcription profiles in reproductive tissues between monoembryonic and polyembryonic varieties. The full length of *CitRKD1* was isolated from satsuma mandarin and its molecular function was investigated using transgenic ‘Hamlin’ sweet orange by antisense-overexpression. The examined citrus varieties contained a pair of *CitRKD1* alleles at the polyembryonic locus. The allele with a MITE insertion in the upstream region was highly expressed in reproductive tissues and is likely involved in somatic embryogenesis. We also developed a poly/monoembryony discriminating DNA marker using the conserved sequences of *CitRKD1* alleles that could help increase genetic diversity in citrus breeding.

## Results

### Microarray analysis to identify the candidate gene regulating citrus somatic embryogenesis

**Fig. 1:**
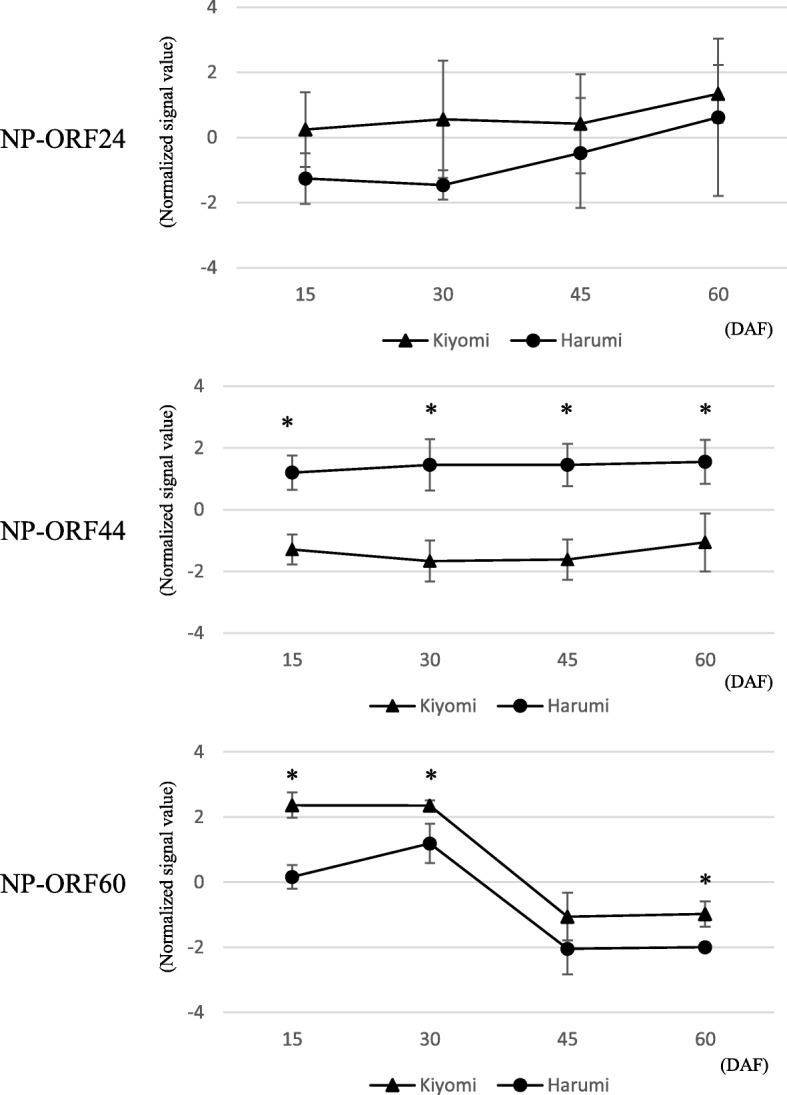
Microarray expression patterns of three selected genes in young whole fruits at 15–60 DAF of ‘Kiyomi’ (monoembryonic variety) and ‘Harumi’ (polyembryonic variety). The expression values of each candidate gene indicate the average normalized expression values of their probes. Each error bar indicates standard deviation among expression values of oligonucleotide probes derived from different exons in the same gene. Samples showing significant differences at *P* < 0.05 between ‘Kiyomi’ and ‘Harumi’ are marked with an asterisk

**Fig. 2:**
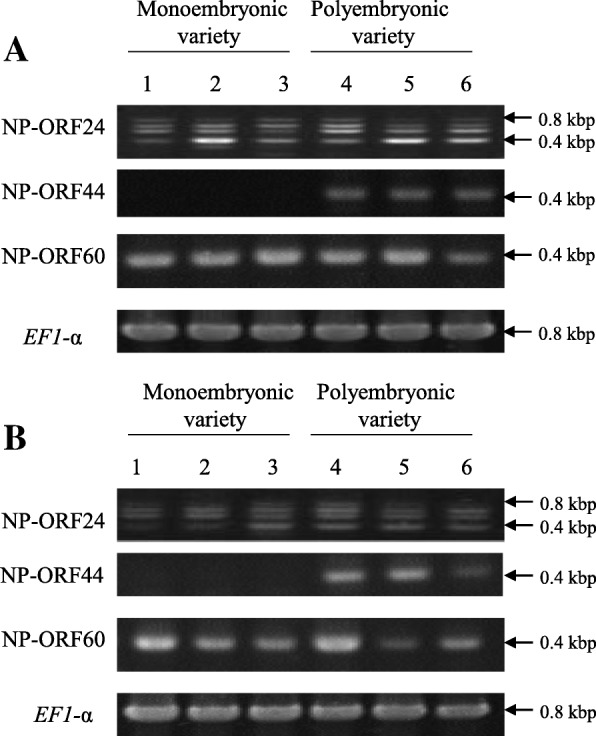
RT-PCR expression patterns of four candidate genes in young whole fruits at 15 DAF (**a**) and at 30 DAF (**b**) of monoembryonic (1–3) and polyembryonic (4–6) varieties. 1: ‘Kiyomi’, 2: clementine mandarin, 3: Mato buntan pumelo, 4: ‘Harumi’, 5: satsuma mandarin, 6: ponkan mandarin. The transcription of NP-ORF44 showed significant differences among monoembryonic and polyembryonic varieties

To identify the principal gene regulating citrus somatic embryogenesis in the polyembryonic locus, a custom oligo-DNA microarray was designed using the draft sequence. In total, 391 probes including multiple probes for each newly predicted ORF (named NP-ORF to discriminate them from previously reported ORFs for the polyembryonic locus [28]) and 29,148 probes for mRNA loci of the clementine mandarin genome were mounted on the custom oligo-DNA microarray, in which 50 genes overlapped between the polyembryonic locus and mRNA loci of the clementine genome sequence. This custom oligo-DNA microarray was used to compare gene expression patterns among young whole fruits at 15, 30, 45 and 60 days after flowering (DAF) of ‘Kiyomi’ (*C. unshiu* Marc*. × C. sinensis* L. Osbeck) as a monoembryonic variety and ‘Harumi’ (‘Kiyomi’ *× C. reticulata* Blanco) as a polyembryonic variety. Under strict filter conditions, 12 of the 391 probes in the polyembryonic locus consistently showed significant expression changes greater than 2-fold between ‘Kiyomi’ and ‘Harumi’ throughout experimental period. These probes were derived from NP-ORF24 (3 probes), NO-ORF44 (6 probes) and NP-ORF60 (3 probes). The expression patterns of these candidate genes are shown in Fig. 1, using the average expression values of their corresponding probes. NP-ORF24 primarily showed homology to ciclev10003992m in scaffold 5 of the clementine mandarin genome sequence, which was a different region from the polyembryonic locus. It was annotated as phosphoribosylaminoimidazole carboxylase but had partially homology to other different loci of clementine mandarin genomes, implying it may be a member of a possible multigene family. The expression values of NP-ORF24 in ‘Kiyomi’ tended to be higher than those in ‘Harumi’ but the standard deviation was very large across the whole experiment. NP-ORF44 showed high homology to Ciclev10010497m, which was annotated as a RWP-RK domain-containing protein. The expression values of NP-ORF44 in ‘Harumi’ were significantly higher than those in ‘Kiyomi’. NP-ORF60 showed high homology to ciclev10009286m, which was annotated as a MYB-like DNA-binding protein. The expression value of NP-ORF60 remained high until 30 DAF and then decreased to its lowest level at 45 DAF, with parallel expression in the two varieties. Reverse transcription-polymerase chain reaction (RT-PCR) was carried out to confirm these expression patterns using cDNA derived from young whole fruits at 15 and 30 DAF of three monoembryonic varieties (‘Kiyomi’, clementine mandarin, Mato buntan pumelo (*Citrus grandis* L. Osbeck)) and three polyembryonic varieties (‘Harumi’, satsuma mandarin, ponkan mandarin) (Fig. 2). Only NP-ORF44 showed a clear difference among them. Various sizes of PCR fragments ranging from 400 bp to 800 bp were observed for NP-ORF24 although PCR fragment was expected to be single and 400 bp in size. This expression pattern was in agreement with a blast search result suggesting that NP-ORF24 might have multiple paralogs on various loci in citrus. According to these results, NP-ORF44 was selected as a candidate for the gene that regulates somatic embryogenesis out of 79 NP-ORFs in the polyembryonic locus region.

**Fig. 3:**
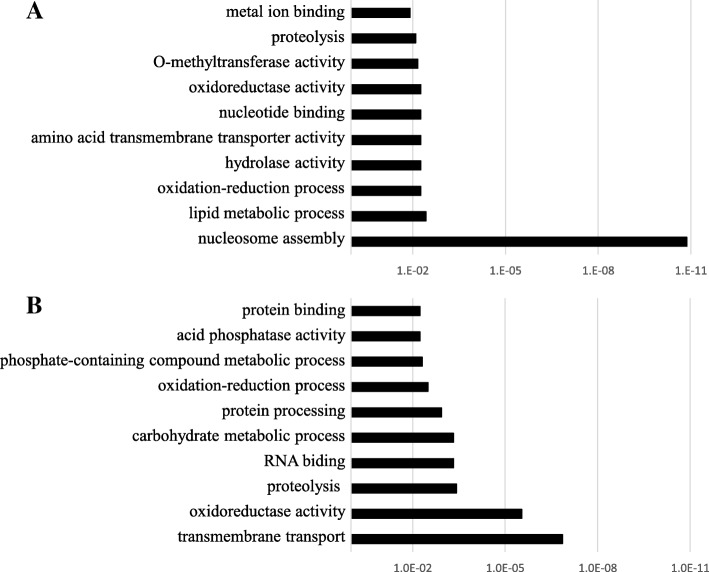
GO term enrichment analysis of highly expressed genes (**a**) and weakly expressed genes (**b**) using 356 genes with more than 2-fold differences in expression ratios between of ‘Kiyomi’ (monoembryonic variety) and ‘Harumi’ (polyembryonic variety) during whole experimental period. The Y-axis indicates the Fisher’s test *P*-value (−log10)

Among the 29,148 probes generated from mRNA sequences of the clementine mandarin genome, 356 genes including NP-ORF44 (Ciclev10010497m) showed more than 2-fold expression changes between ‘Kiyomi’ and ‘Harumi’ throughout the experimental period (Additional file 1: Table S1 and Additional file 2: Table S2). The expression of 85 genes was highly expressed while 270 genes were weakly expressed in reproductive tissues of ‘Harumi’ in comparison with those of ‘Kiyomi’. Among them were several genes commonly identified in past reports that are likely associated with somatic embryogenesis, such as UDP-glycosyltransferase superfamily proteins, ankyrin repeat family proteins, heat shock proteins, and protein kinases [22, 23, 27]. These genes were reported to be involved in oxidative stress responses and callose deposition in the cell. GO term enrichment analysis was carried out to interpret their biological functions (Fig. 3)*.* Nucleosome assembly was the most enriched term in polyembryonic reproductive tissues, followed by lipid metabolic process, oxidation-reduction process, and hydrolase activity. Conversely, transmembrane transport, oxidoreductase activity, proteolysis, and RNA binding processes were less active. Although some of these terms might reflect differences of genome composition between ‘Harumi’ and ‘Kiyomi’, this result indicated that polyembryonic reproductive tissues progress more active cell proliferation than monoembryonic reproductive tissues. In fact, the growth of somatic embryos is generally precocious and more vigorous than that of zygotic embryos. Oxidation reduction-related GO terms were found in both lists but the frequency was comparatively higher among the weakly expressed genes. In cotton (*Gossypium hirsutum* L.), inducing oxidative stress promoted somatic embryogenesis [9]. Oxidation reduction-related GO terms were extremely enriched during the early bud stage before flowering [22, 23].

### Isolation and sequence analysis of the cDNA and genomic clone of CitRKD1

**Fig. 4:**
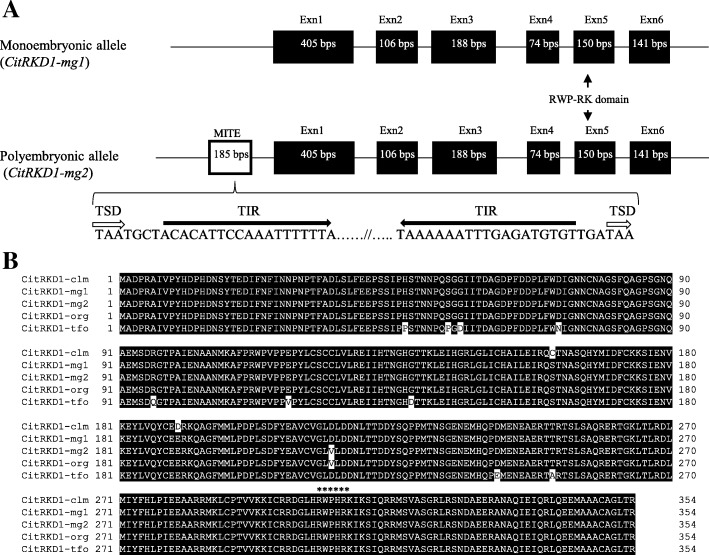
Genomic structure of *CitRKD1* alleles (*CitRKD1*-*mg1* and *CitRKD1-mg2*) isolated from satsuma mandarin (**a**) and amino acid sequence alignment (**b**) of *CitRKD1* alleles of orange (*CitRKD1-org*, orange1.1g041600m), clementine mandarin (*CitRKD1-clm*, Ciclev10010497m) and trifoliate orange (*CitRKD1-tfo*, ANH22496) with those of satsuma mandarin. A miniature inverted-repeat transposable element (MITE) comprising the typical target site duplication (TSD) and terminal inserted repeat (TIR) structure was found in the upstream region of *CitRKD1-mg2.* The asterisks indicate the conserved RWP-RK domain in plant RKD genes

Full-length cDNA and genomic clones corresponding to NP-ORF44 were isolated from satsuma mandarin and their genomic structures were characterized (Fig. 4a). Two independent RKD homologs, named *CitRKD1-mg1* and *CitRKD1-mg2*, each comprised a 1065 bp ORF encoding 354 deduced amino acids with a molecular mass of 86.8 kDa and a pI of 4.2. The ORFs had six nucleotide differences between them but most were synonymous mutations, with only one non-synonymous mutation. Their genomic clones had six exons and five introns, and a 219 bp sequence including a 185 bp putative MITE sequence was inserted in the upstream region of *CitRKD1-mg2*. The MITE had a typical structure with a target site duplication (TSD) and terminal inserted repeat (TIR). The insertion was flanked by a 3 bp TSD (TAA) and the ends of the element had a 19 bp TSD (ACACATTCCAAATTTTTTA). BLAST analysis revealed that these genes were highly homologous to RKD genes of sweet orange (orange1.1g041600m), clementine mandarin (Ciclev10010497m) and trifoliate orange (*Poncirus trifoliata* Raf.) (ANH22496) with more than 97.5% identity at the amino acid sequence level. These citrus RKD genes were named *CitRKD1* and the RKD genes of sweet orange (*CitRKD1-org*), clementine mandarin (*CitRKD1-clm*) and trifoliate orange (*CitRKD1-tfo*) were considered as alleles of *CitRKD1*. The amino acid sequences of *CiRKD1-mg1* and *CitRKD1-mg2* were aligned with other citrus *CitRKD1* alleles (Fig. 4b). Their deduced amino acid sequences contained typical RWP-RK domains in the carboxy terminal region. RKD family members containing the RWP-RK domain have recently been characterized as important regulators of an egg cell-related gene expression program [35]. The RWP-RK domain consists of a basic region, helix region and loop, and the amino acid sequences around these regions were well conserved among the citrus species. Because the amino acid sequences of the citrus *CitRKD1* alleles were highly conserved, their protein functions are expected to be almost identical.

**Fig. 5:**
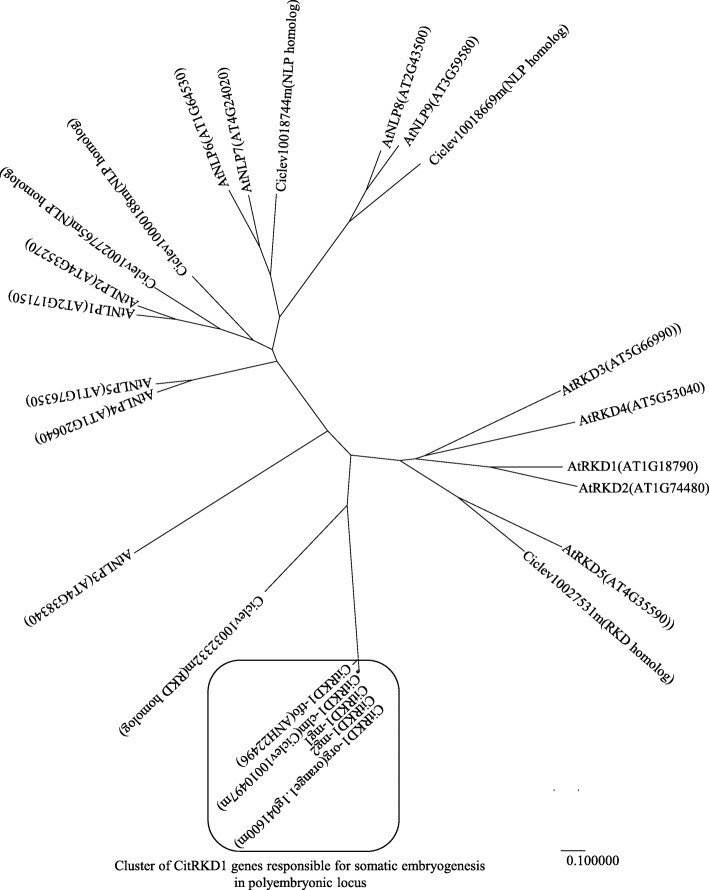
Phylogenetic tree analysis of RWP-RK domain-containing proteins in *Arabidopsis* and *Citrus* by the neighbor-joining (NJ) method. The tree is divided into two major nodes of NLPs and RKDs. NLP: NIN-like protein, RKD: RWP-RK domain-containing protein. There are three RKD homologs derived from different loci in the clementine mandarin genome (Ciclev10010497m in scaffold 1, Ciclev10032332m in scaffold 4, Ciclev10027531m in scaffold 7). The *Citrus CitRKD1* alleles were clustered with *CitRKD1-clm* (Ciclev10010497m) in scaffold 1, which corresponds to the polyembryonic locus controlling embryonic type

Neighbour-joining phylogenetic tree analysis was carried out using the amino acid sequences of the *CitRKD1* alleles, and NLP and RKD genes of clementine mandarin and *Arabidopsis* (Fig. 5). There are seven RWP-RK domain-containing proteins in the clementine mandarin genome. Ciclev10010497m in scaffold 1, Ciclev10032332m in scaffold 4, and Ciclev10027531m in scaffold 7 are structurally classified into the RKD gene family. The citrus *CitRKD1* alleles were clustered with Ciclev10010497m in scaffold 1, which corresponded to the polyembryonic locus controlling embryonic type, implying that *CitRKD1* comprised multiple alleles at this locus. Because the *CitRKD1* alleles clustered more closely with *Arabidopsis* RKD genes than *Arabidopsis* NLP genes, *CitRKD1* was considered a member of the RKD family and should play a principal role in regulating somatic embryogenesis.

### Expression analysis of CitRKD1 in various tissues of Satsuma mandarin by RT-PCR

**Fig. 6:**
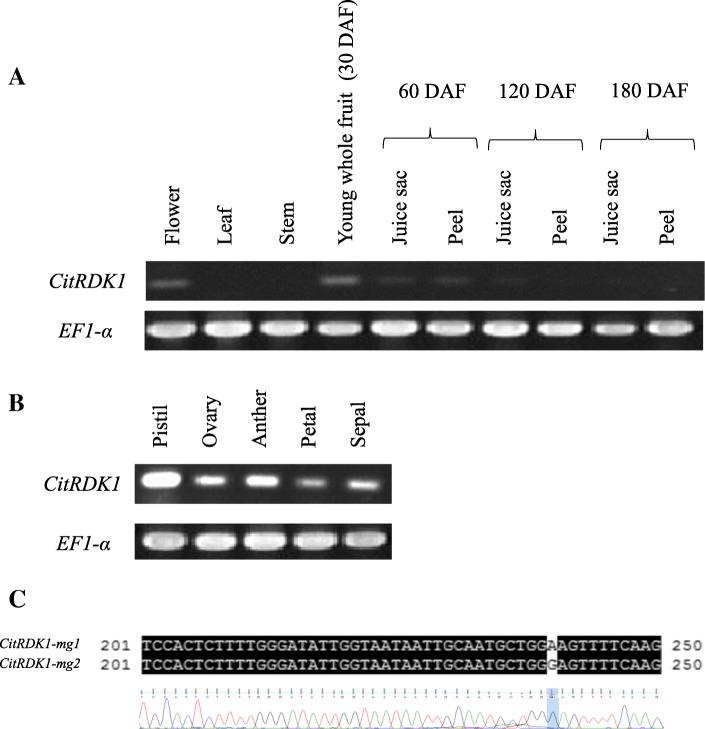
Gene expression patterns of *CitRKD1* in the flower, leaf, stem, young whole fruit at 30 days after flowering (DAF), and fruit tissues (juice sac and peel) from 60 to 180 DAF (**a**), and floral organs at anthesis (**b**) of satsuma mandarin by RT-PCR. *EF1-α* was used as an endogenous control gene. The PCR fragments were sequenced (**c**) and the *CitRKD1* transcripts were confirmed to derive from *CitRDK1-mg2* based on a SNP

RT-PCR was carried out to investigate the expression pattern of *CitRKD1* in various tissues of satsuma mandarin. *CitRKD1* transcripts were detected in flowers at anthesis and young whole fruit at 30 DAF; thereafter, the transcript level decreased towards 60 DAF (Fig. 6a). In floral organs at anthesis, *CitRKD1* transcripts were detected in all examined tissues including the pistil, ovary, anther, petal and sepal (Fig. 6b). In contrast, no transcripts were detected in the vegetative tissues of leaves and stems, or in mature fruit. This expression pattern is likely associated with somatic embryo development in the seed, in which the formation of a primordium cell and initial spherical embryo was observed in the flowering bud stage and around 60 DAF, respectively [18, 19].

Satsuma mandarin had two alleles (*CitRKD1-mg1* and *CitRKD1-mg2*) for *CitRKD1*, but conventional RT-PCR could not clarify whether the obtained PCR fragments came from either or both. Direct sequencing analysis of the PCR fragments indicated that transcripts were amplified from *CitRKD1-mg2* based on a SNP (A or G) at the 240th nucleotide from the initiation position of the coding region (Fig. 6c). The transcription of *CitRKD1-mg2* with a MITE insertion in the upstream region would be responsible for somatic embryogenesis.

### Production of transgenic sweet orange transformed with CitRKD1-mg2 in the antisense direction

**Fig. 7:**
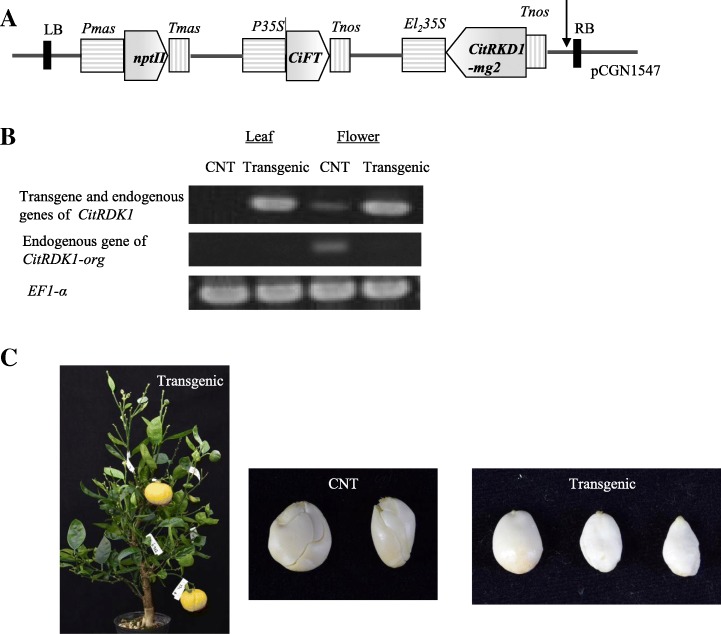
Structure of the *CiFT* co-expression vector with antisense *CitRKD1-mg2* (**a**) and gene expression of *CitRKD1* in leaves and flowers of control ‘Hamlin’ sweet orange (CNT) and transgenic sweet orange by RT-PCR (**b**). The transcription level of endogenous *CitRKD1-org* is investigated using *CitRKD1-org* specific primers. Transgenic sweet orange bearing fruits, seeds of control and transgenic ‘Hamlin’ sweet oranges after removing their seed coats are photographed (**c**). Only one independent line of regenerated transgenic sweet orange was obtained out of 1274 *Agrobacterium* infected epicotyl segments which are generally sufficient number to obtained plural independent transgenic lines, probably owing to deleterious effects of transgene

To identify the function of *CitRKD1*, the coding region of *CitRKD1-mg2* in the antisense direction was inserted into the *CiFT* co-expression vector (Fig. 7a) [7]. ‘Hamlin’ sweet orange has polyembryonic seeds in nature and it was expected that transgenic ‘Hamlin’ sweet orange would fail to produce polyembryonic seeds*.* A total of 1274 epicotyl segments from ‘Hamlin’ sweet orange seedlings were transformed with *Agrobacterium* carrying *CiFT* co-expression vector construct, which were usually sufficient number to obtain plural independent transgenic lines. Ultimately, only one independent transgenic line was recovered, probably owing to poor regeneration and growth, and was subsequently grown in the greenhouse. Integration of the transgene into ‘Hamlin’ sweet orange was investigated through PCR analysis and the obtained transgenic line was confirmed to carry the vector construct. Southern blot analysis was carried out to determine the copy number of the transgene in the transgenic line, and revealed that it had a single copy (data not shown). RT-PCR analysis revealed that transcripts of the transgene were highly accumulated in leaves and flowers in the transgenic line (Fig. 7b). In control ‘Hamlin’ sweet orange, transcripts of the endogenous sweet orange *CitRKD1* allele (*CitRKD1-org*) were not detected in leaves and were accumulated in flowers. RT-PCR using *CitRKD1-org* specific primers revealed that the transcription level of endogenous *CitRKD1-org* was reduced under detection level in the transgenic sweet orange by the effect of transgene. For 1.5 years after *Agrobacterium* infection, morphologically normal flowers with fertile pollen bloomed but fruit set failed. Thereafter, transgenic sweet orange succeeded to bear fruits in 3 years after infection and self-pollinated mature fruits were harvested at 180 DAF.

### Loss of CitRKD1 function by antisense-overexpression resulted in failure to generate polyembryonic seeds in transgenic sweet orange

**Fig. 8:**
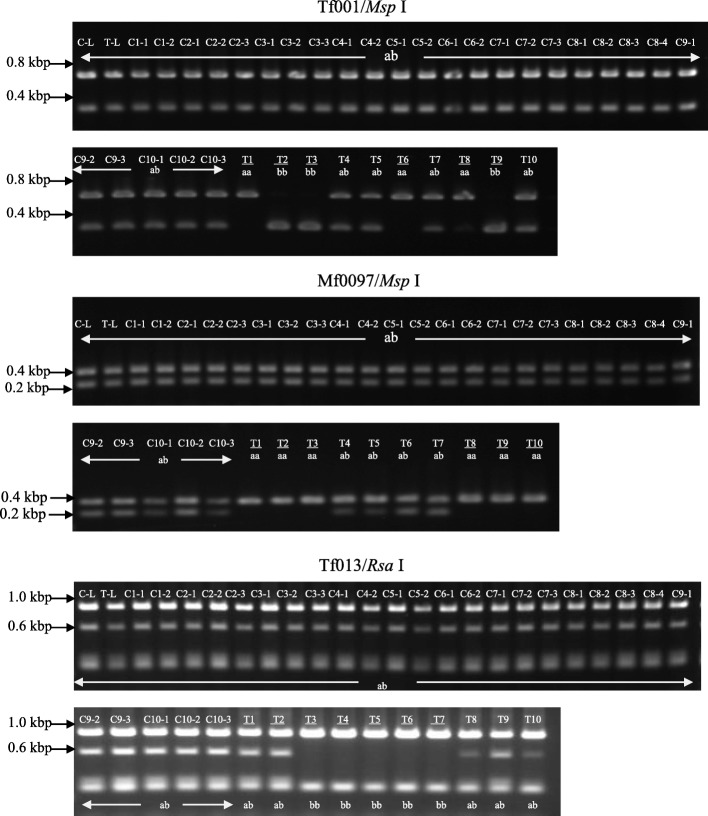
CAPS marker analysis of T_1_ plants germinated from seeds of control sweet orange (C1–1 to C10–3) and transgenic sweet orange (T1 to T10). DNA isolated from wild type sweet orange leaves (C-L) and transgenic sweet orange leaves (T-L) was used as a reference template. T_1_ plants with an underline had different genotypes from the control sweet orange and mother tree. Identical genotypes to sweet orange for the three CAPS markers means the T_1_ plant germinated from a polyembryo, while a different genotype means the T_1_ plant germinated from a zygotic embryo produced by self-pollination

The seed coats were removed from the seeds of harvested transgenic fruits, and transgenic and control ‘Hamlin’ sweet orange seeds were subsequently photographed (Fig. 7c). In transgenic sweet orange, most seeds exhibited a morphologically smooth surface and a few seeds had a slightly rough surface. In contrast, all seeds of the control ‘Hamlin’ sweet orange were recognizable as polyembryonic seeds at a glance. In the control sweet orange, 2–4 independent T_1_ plants germinated from each seed and a total of 27 independent T_1_ plants were grown from 10 seeds in pots. Conversely, 10 independent T_1_ plants were grown from 10 seeds of transgenic sweet orange. Cleaved amplified polymorphic sequence (CAPS) marker analysis was carried to investigate whether the T_1_ plants germinated from self-pollinated zygotic embryos or somatic embryos. Five CAPS markers located on five different linkage groups on the AGI map [36], which exhibited heterozygous genotypes in sweet orange, were used for DNA diagnosis of T_1_ plants. All T_1_ plants germinated from the control sweet orange had the same heterozygous genotype (a/b) for the five CAPS markers examined as the sweet orange and transgenic mother trees. In contrast, all T_1_ plants germinated from the transgenic sweet orange seeds had different genotypes to the mother tree (a/a or b/b), which occurred by self-pollination, for either of the five CAPS markers. A minimum set of three CAPS markers (Tf0001/*Msp*I, Mf0097/*Dra*I, Tf0013/*Rsa*I) could genetically discriminate all transgenic T_1_ plants from the mother tree (Fig. 8). This confirmed that the loss of *CitRKD1* function from antisense-overexpression resulted in failure to generate polyembryonic seeds in transgenic sweet orange. This result provided strong genetic evidence that *CitRKD1* plays an important role in regulating citrus somatic embryogenesis.

### Association analysis between the MITE insertion in the upstream region and transcription of the CitRKD1 alleles

**Fig. 9:**
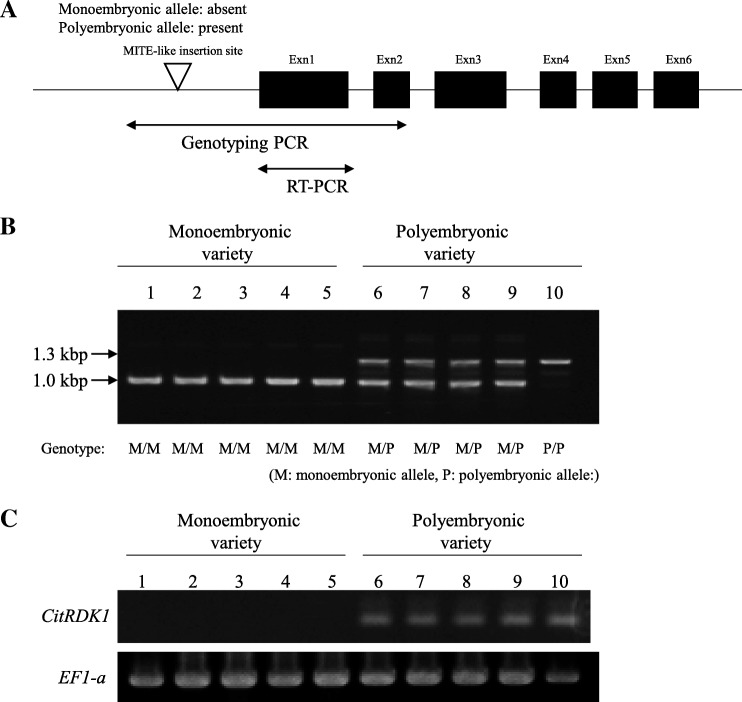
Association between promoter structure and transcription of *CitRDK1* in 10 representative citrus varieties. The illustration (**a**) indicates the PCR amplification region of *CitRDK1* in genotyping PCR (**b**) and RT-PCR (**c**). RT-PCR was conducted using young whole fruit at DAF30. The genotypes of *CitRKD1* alleles are shown by the combinations of M (monoembryonic allele) and P (polyembryonic allele). 1: clementine mandarin, 2: ‘Kiyomi’, 3: Mato buntan pumelo, 4: hyuga-natsu, 5: ‘Nishinokaori’ tangor, 6: satsuma mandarin, 7: ‘Harumi’, 8: ‘Valencia’ sweet orange, 9: ponkan mandarin, 10: ‘Saga’ mandarin

Of the two *CitRKD1* alleles in satsuma mandarin, transcription was only observed for the allele with the MITE insertion in the upstream region. To understand the association between the MITE insertion in the upstream region and the transcription of *CiRKD1* alleles, a tentative genotyping PCR assay was carried out to amplify the genomic region around the MITE insertion site (Fig. 9a). The five monoembryonic varieties commonly had an approximately 1.0 kbp single genomic PCR fragment, while the five polyembryonic varieties commonly had a 1.3 kbp genomic PCR fragment with or without a 1.0 kbp fragment (Fig. 9b). The 1.0 kbp genomic PCR fragment was derived from *CitRKD1* without the MITE insertion and the 1.3 kbp genomic PCR fragment came from *CitRKD1* with the MITE insertion. The monoembryonic varieties had homozygous genotypes for the *CitRKD1* allele without the MITE insertion, while the polyembryonic varieties had heterozygous genotypes with and without the MITE insertion, or homozygous genotypes with the MITE insertion. RT-PCR analysis revealed that transcripts were only present in the reproductive tissues of the polyembryonic varieties (Fig. 9c). Sequence analysis of the PCR fragments amplified from genomic DNA and cDNA indicated that the transcripts were generated from the alleles with the MITE insertion (data not shown). Therefore, *CitRKD1* alleles without the MITE insertion could be designated monoembryonic alleles and *CitRKD1* alleles with the MITE insertion were designated polyembryonic alleles. Considering that *CitRKD1* transcripts were observed only in polyembryonic varieties with polyembryonic alleles, it is conceivable that the MITE insertion in the upstream region might affect the transcription level of *CitRKD1*.

### Consistency between allelic genotypes of CitRKD1 and embryonic types in 95 traditional and breeding varieties

**Fig. 10:**
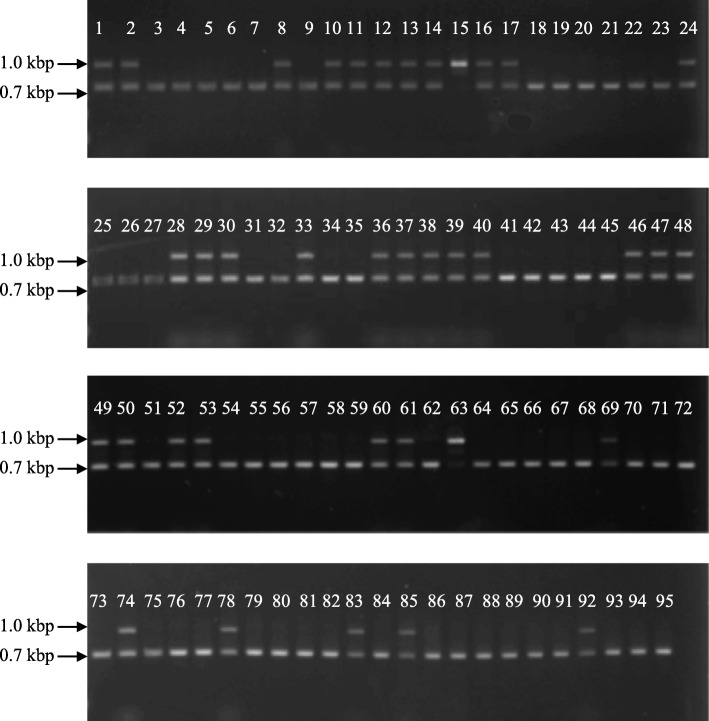
Allelic genotyping PCR of *CitRDK1* alleles for 95 traditional and breeding varieties in Japanese breeding programs using the P/M marker. The upper fragment is derived from the polyembryonic allele and the lower fragment is derived from the monoembryonic allele. ‘Seminole’ (15) and ‘Saga’ mandarin (63) possess homozygous polyembryonic alleles in their genomes

To confirm the consistency between *CitRKD1* allelic genotypes and embryonic phenotypes, the genotypes of 95 traditional and breeding varieties from Japanese breeding programs were investigated for MITE insertion allelism. A pair of primers was newly designed using the conserved sequences among *CitRKD1* alleles of 14 ancestral varieties from Japanese breeding programs (Table 1) [14]. In allelic genotyping PCR using the new primer set, approximately 0.7 and 1.0 kbp fragments corresponded to monoembryonic alleles (M) and polyembryonic alleles (P), respectively (Fig. 10). Among the 14 ancestral varieties, Duncan grapefruit, Dancy tangerine (*C. tangerina* hort. ex Tanaka), sweet orange, satsuma mandarin, ponkan mandarin, Mediterranean mandarin (*C. deliciosa* Ten.), king mandarin (*C. nobilis* Lour.) and ‘Murcott’ showed approximately 0.7 and 1.0 kbp fragments, indicating M/P genotype, while clementine mandarin, ‘Mukaku-kishu’ (*C. kinokuni* hort. ex Tanaka), Mato buntan pumelo, hassaku (*C. hassaku* hort. ex Tanaka), hyuga-natsu (*C. tamurana* hort. ex Tanaka) and iyo-kan (*C. iyo* hort. ex Tanaka) had single fragments of approximately 0.7 kbp, indicating M/M genotype. Based on the fragment patterns of the allelic genotyping PCR, allelic genotyping of *CitRKD1* was performed for the remaining 81 varieties. The genotypes of 52 varieties were M/M and those of 27 pedigrees were M/P. Only ‘Seminole’ and ‘Saga’ mandarin had P/P genotype, with single fragments of approximately 1.0 kbp in allelic genotyping PCR. The embryonic types of the 95 varieties were perfectly consistent with their allelic genotypes, with M/M varieties having monoembryonic seeds and M/P or P/P varieties having polyembryonic seeds. This allelic genotype and embryonic type information is summarized in Table 1.

**Table 1:** Embryonic phenotype and allelic genotype of *CitRDK1* for 95 traditional and breeding varieties in Japanese breeding program

Code	Variety	Academic name	Parental combination	Embryounic phenotype	Allelic genotype
1	grapefruit	*C. paradisi Macfad.C. paradisi Macfad.C. paradisi Macfad.*	Ancestral variety	polyembryonic	M/P
2	dancy tangerine	*C. tangerina hort. ex TanakaC. tangerina hort. ex TanakaC. tangerina hort. ex Tanaka*	Ancestral variety	polyembryonic	M/P
3	clementine mandarin	*C. clementina hort. ex TanakaC. clementina hort. ex TanakaC. clementina hort. ex Tanaka*	Ancestral variety	monoembryonic	M/M
4	Mukaku-kishu	*C. kinokuni hort. ex TanakaC. kinokuni hort. ex TanakaC. kinokuni hort. ex Tanaka*	Ancestral variety	monoembryonic^a^	M/M
5	Tanigawa-buntan	*C.grandis OsbeckC.grandis OsbeckC.grandis Osbeck*	Ancestral variety	monoembryonic	M/M
6	hassaku	*C. hassaku hort. ex TanakaC. hassaku hort. ex TanakaC. hassaku hort. ex Tanaka*	Ancestral variety	monoembryonic	M/M
7	hyuga-natsu	*C. tamurana hort. ex TanakaC. tamurana hort. ex TanakaC. tamurana hort. ex Tanaka*	Ancestral variety	monoembryonic	M/M
8	sweet orange	*C. sinensis (L.) OsbeckC. sinensis (L.) OsbeckC. sinensis (L.) Osbeck*	Ancestral variety	polyembryonic	M/P
9	Iyo-kan	*C. iyo hort. ex TanakaC. iyo hort. ex TanakaC. iyo hort. ex Tanaka*	Ancestral variety	monoembryonic	M/M
10	satsuma mandarin	*C. unshiu Marc.C. unshiu Marc.C. unshiu Marc.*	Ancestral variety	polyembryonic	M/P
11	ponkan mandarin	*C. reticulata BlancoC. reticulata BlancoC. reticulata Blanco*	Ancestral variety	polyembryonic	M/P
12	mediterranean mandarin	*C. deliciosa Ten.C. deliciosa Ten.C. deliciosa Ten.*	Ancestral variety	polyembryonic	M/P
13	king mandarin	*C. nobilis* Lour.C. nobilis Lour.C. nobilis Lour.	Ancestral variety	polyembryonic	M/P
14	Murcott	*–*	Ancestral variety	polyembryonic	M/P
15	Seminole	*–*	1 × 2	polyembryonic	P/P
16	Minneola	*–*	1 × 2	polyembryonic	M/P
17	Orlando	*–*	1 × 2	polyembryonic	M/P
18	Fortune	*–*	3 × 17	monoembryonic	M/M
19	Southern yellow	*–*	5 × 4	monoembryonic^a^	M/M
20	Nankou	*–*	10 × 3	monoembryonic	M/M
21	Ariake	*–*	8 × 3	monoembryonic	M/M
22	Sweet spring	*–*	10 × 6	monoembryonic	M/M
23	JHG	*–*	10 × 7	monoembryonic	M/M
24	Awa orange	*–*	7 × 8	polyembryonic	M/P
25	Kiyomi	*–*	10 × 8	monoembryonic	M/M
26	Aki tangor	*–*	10 × 8	monoembryonic	M/M
27	HF9	*–*	10 × 8	monoembryonic	M/M
28	Kankitsu chukanbohon Nou 6 gou	*–*	13 × 4	polyembryonic^a^	M/P
29	Hayaka	*–*	10 × 11	polyembryonic	M/P
30	Kara mandarin	*–*	10 × 13	polyembryonic	M/P
31	Encore	*–*	13 × 12	monoembryonic	M/M
32	Wilking	*–*	13 × 12	monoembryonic	M/M
33	Page	*–*	3 × 16	polyembryonic	M/P
34	Robinson	*–*	3 × 17	monoembryonic	M/M
35	Lee	*–*	3 × 17	monoembryonic	M/M
36	Fairchild	*–*	3 × 17	polyembryonic	M/P
37	Nova	*–*	3 × 17	polyembryonic	M/P
38	Osceola tangerine	*–*	3 × 17	polyembryonic	M/P
39	Seihou	*–*	10 × 16	polyembryonic	M/P
40	Akemi	*–*	10 × 15	polyembryonic	M/P
41	A7	*–*	22 × 8	monoembryonic	M/M
42	Okitsu 46 gou	*–*	22 × 8	monoembryonic	M/M
43	Nishino kaori	*–*	25 × 8	monoembryonic	M/M
44	KyOw No.21	*–*	25 × 10	monoembryonic	M/M
45	KyOw14	*–*	25 × 10	monoembryonic	M/M
46	Tsunokaori	*–*	25 × 10	polyembryonic	M/P
47	Shiranui	*–*	25 × 11	polyembryonic	M/P
48	Youkou	*–*	25 × 11	polyembryonic	M/P
49	Harumi	*–*	25 × 11	polyembryonic	M/P
50	Setomi	*–*	25 × 11	polyembryonic	M/P
51	EnOw21	*–*	31 × 10	monoembryonic	M/M
52	Kuchinotsu 39 gou	*–*	31 × 10	polyembryonic	M/P
53	Mihokoru	*–*	10 × 31	polyembryonic	M/P
54	KyEn No.4	*–*	25 × 31	monoembryonic	M/M
55	Tsunonozomi	*–*	25 × 31	monoembryonic	M/M
56	Amaka	*–*	25 × 31	monoembryonic	M/M
57	Okitsu 45 gou	*–*	25 × 32	monoembryonic	M/M
58	Tamami	*–*	25 × 32	monoembryonic	M/M
59	Benibae	*–*	27 × 31	monoembryonic	M/M
60	Hareyaka	*–*	31 × 11	polyembryonic	M/P
61	Amakusa	*–*	45 × 33	polyembryonic	M/P
62	Kuchinotsu 38 gou	*–*	44 × 34	monoembryonic	M/M
63	Saga mandarin	*–*	10 × 36	polyembryonic	P/P
64	Kankitsu chukanbohon Nou 5 gou	*–*	35 × 4	monoembryonic^a^	M/M
65	E-647	*–*	25 × 38	monoembryonic	M/M
66	Southern Red	*–*	30 × 38	monoembryonic	M/M
67	KyOw No.21·D No.4KyOw No.21·D No.4KyOw No.21·D No.4	*–*	44 × 2	monoembryonic	M/M
68	Kuchinotsu 28 gou	*–*	44 × 2	monoembryonic	M/M
69	Haruhi	*–*	42 × 24	polyembryonic	M/P
70	2700·Oiy-252,700·Oiy-252,700·Oiy-25	*–*	43 × 9	monoembryonic	M/M
71	No.1408	*–*	51 × (25 × 9)	monoembryonic	M/M
72	Kuchinotsu 18 gou	*–*	44 × 31	monoembryonic	M/M
73	Kuchinotsu 35 gou	*–*	44 × 31	monoembryonic	M/M
74	Kanpei	*–*	43 × 11	polyembryonic	M/P
75	Okitsu 57 gou	*–*	42 × 49	monoembryonic	M/M
76	Asumi	*–*	42 × 49	monoembryonic	M/M
77	Asuki	*–*	42 × 49	monoembryonic	M/M
78	Seinannohikari	*–*	51 × 48	polyembryonic	M/P
79	Kuchinotsu 27 gou	*–*	51 × 48	monoembryonic	M/M
80	Okitsu 59 gou	*–*	41 × (25 × 32)41 × (25 × 32)	monoembryonic	M/M
81	Kuchinotsu 33 gou	*–*	45 × 31	monoembryonic	M/M
82	Tsunokagayaki	*–*	45 × 31	monoembryonic	M/M
83	Setoka	*–*	54 × 14	polyembryonic	M/P
84	Kuchinotsu 36 gou	*–*	? × 14	monoembryonic	M/M
85	Reikou	*–*	? × 14	polyembryonic	M/P
86	Ehime kashi No. 28 gou	*–*	20 × 61	monoembryonic	M/M
87	Kuchinotsu 49 gou	*–*	(KO21 ‘ ROb16) ‘ 71(KO21 ‘ ROb16) ‘ 71	monoembryonic	M/M
88	Okitsu 56 gou	*–*	57 × 64	monoembryonic	M/M
89	No.1011	*–*	20 × 70	monoembryonic	M/M
90	Kuchinotsu 51 gou	*–*	(? × 2) × 79(? × 2) × 79	monoembryonic	M/M
91	Harehime	*–*	65 × 10	monoembryonic	M/M
92	Okitsu 63 gou	*–*	65 × 11	polyembryonic	M/P
93	Kuchinotsu 52 gou	*–*	82 × 21	monoembryonic	M/M
94	Mihaya	*–*	55 × 71	monoembryonic	M/M
95	Okitsu 67 gou	*–*	91 × 88	monoembryonic	M/M

*M* monoembryonic allele, *P* polyemrbyonic allele

^a^: Phenotype is not visually confimed due to seedless but is determined based on the phenotypes of pedigrees in the cross hybrid tests

Parent-offspring trio analysis was carried out to validate the allele combinations inherited from parent varieties. The allele genotypes of most parent varieties were M/M or M/P, so their offspring genotypes should be either M/M or M/P but not P/P. Among the 78 parent-offspring combinations in which parentage was confirmed by DNA diagnosis [31], no discrepancies were observed in the parent-offspring relationships. The polyembryonic alleles of ‘Seminole’ and ‘Saga’ were inherited from polyembryonic alleles in both parents with the M/P genotype. These results confirmed that MITE insertion was correlated with polyembryonic seed development and that the polyembryonic allele was dominant against the monoembryonic allele.

## Discussion

In the present study, we report the isolation and functional characterization of *CitRKD1*, which has a RWP-RK domain and is likely involved in somatic embryogenesis in citrus nucellar tissues. *CitRKD1*, which corresponds to NP-ORF44 at the polyembryonic locus controlling embryonic type, was identified to regulate somatic embryogenesis in nucellar tissues through various analyses of gene expression, genomic structure and loss of function using transgenic sweet orange. Plant-specific RWP-RK family transcription factors are phylogenetically classified into two major subfamilies: NIN-like proteins (NLPs), which are key regulators of N signalling and are involved in nodule organogenesis, and RKD proteins, which are involved in egg cell specification and differentiation [5]. Most RKD genes are expressed predominantly in plant reproductive tissues and are considered to regulate egg cell proliferation by reprograming nucellar cells. *AtRKD5* has a broad expression profile throughout all *Arabidopsis* tissues including vegetative organs and might have a fundamental function during the plant life cycle rather than in egg cell formation [21, 34]. Phylogenetic analysis (Fig. 5) suggested the citrus RKD genes responsible for somatic embryogenesis might have functionally diverged from *Arabidopsis* RKD genes from a putative common ancestor gene during the evolutionary process in dicotyledonous plants. *CitRKD1* lacked motif 12 of the RKD(A) subfamily structure but possessed motifs 1 and 2 around the RWP-RK domain [5]. Therefore, CitRKD1 would be expected to have a similar protein function in regulating egg cell proliferation by reprograming nucellar cells.

The effect of loss of *CitRKD1* function on somatic embryogenesis was investigated using transgenic sweet orange by antisense-overexpression and resulted in the failure of somatic embryogenesis. Although only one independent line of transgenic sweet orange was generated in this study, this result provides important genetic evidence that *CitRKD1* at the polyembryonic locus is involved in somatic embryogenesis and that the transcription of *CitRKD1* is required to generate polyembryos in nucellar tissues. At present, it is still hard to evaluate reproductive organ traits in transgenic perennial fruit trees because of their long juvenile stage. In addition, a possible reason for the low success rate of transgenic lines might be deleterious effects of the transgene. In *Arabidopsis*, mutants of the *AtRKD1* and *AtRKD2* genes responsible for egg cell proliferation did not display obvious defects in either sporophytic or gametophytic tissues due to functional redundancy within the *AtRKD* gene family, but ectopic expression of *AtRKD1* and *AtRKD2* caused severe distortion of plant growth including aberrant tissue proliferation [21]. In addition, *Atrkd4* loss of function mutants showed impaired zygotic cell elongation and subsequent cell division patterning [40]. In *Marchantia polymorpha*, *MpRKD*, which is orthologous to *AtRKD* genes, is required to keep the egg cell quiescent in the absence of fertilization by preventing parthenogenesis, but ectopic expression of *MpRKD* in *Arabidopsis* did not produce any obvious phenotypes [34]. Considering the RDK family genes are intimately intertwined with other RDK family genes or with downstream associated genes in the embryogenic process, it is possible that the loss of *CitRKD1* function might have deleterious effects on the regeneration of sweet orange. In fact, the feasibility of obtaining regenerated plants in citrus depends on the maternal genotype and monoembryonic varieties tend to have lower embryogenic capacity both in vivo and in vitro than polyembryonic varieties [4].

In citrus, multiple alleles of *CitRKD1* with high sequence homologies are located at the polyembryonic locus controlling embryonic type (poly/monoembryony). These alleles were classified structurally into polyembryonic alleles with the MITE insertion and monoembryonic alleles without it. *CitRKD1* transcripts were only detected in the reproductive tissues of polyembryonic varieties with polyembryonic alleles. Therefore, the MITE insertion in the upstream region likely acts as a regulator by affecting transcription. It was reported that the MITE insertion in the upstream region co-segregated with poly/monoembryonic phenotypes in a segregating population and representative varieties [41]. MITE is a class of DNA transposon and comprises TIRs flanked by small direct repeats [8]. MITEs are frequently inserted into promoters, untranslated regions, introns or coding region of plant genes and play an important role in regulating gene expression and in genome evolution. The effects of MITEs inserted close to genes on transcription levels vary, e.g. some MITE insertions increase expression, some decrease expression, and some do not affect expression at all in rice (*Oryza sativa*) [24]. There are many reports that MITE insertion in the upstream region of a gene enhances transcription and results in a new phenotype, such as in *Hordeum vulgare* [38] and *Sorghum bicolor* [26]. The *cis*-regulatory elements in the upstream regions of *CitRKD1-mg1* and *CitRKD1-mg2* were compared using the PLACE database (https://sogo.dna.affrc.go.jp/cgi-bin/sogo.cgi?lang=en&pj=640&action=page&page=newplace**)** (data not shown). Various *cis*-regulatory elements such as a MYB recognition site found in *Arabidopsis* rd22 (MYB2CONSENSUSAT) [1], a binding site for BELL homeodomain transcription factors (BIHD1OS) [25], a copper-response element (CURECORECR) [33], and a light-induced motif (SORLIP1) [12] were commonly found in both upstream regions. While, several *cis*-regulatory elements specific to *CitRKD1-mg2* were found such as a *cis*-regulatory element for L1 layer-specific gene expression (SORLIP1AT9) [2] and a sugar response element (SRE) [39]. These *cis*-regulatory elements are not sufficient to explain the broad expression profile of *CitRKD1* in reproductive tissues of polyembryonic varieties. Considering that *AtRKD1* and *AtRKD2* are temporally and preferentially expressed in egg cells in *Arabidopsis*, it is conceivable that the MITE insertion might alter the innate time- and tissue-specific expression profile in embryogenesis, resulting in broad expression in various reproductive tissues and prolonged gene expression from flowering to the early stage of fruit development.

A DNA marker to detect MITE insertion in the upstream region of *CitRKD1* was newly developed using the conserved sequence among 14 ancestral varieties from Japanese citrus breeding programs. This DNA marker named as P/M marker could be applied to various citrus species including mandarin, sweet orange, pumelo, and their hybrids. The allelic genotypes of 95 varieties and breeding pedigrees evaluated using the P/M marker perfectly matched their embryonic phenotypes and the allele combinations in parent-offspring relationships. These results are in agreement with previous reports that polyembryony in citrus is regulated by a single dominant gene [15, 32]. In citrus, the polyembryony phenomenon makes it difficult to obtain zygotic embryos and is one of the limiting factors to expanding the genetic diversity. The newly developed P/M maker can be applied to a wide range of citrus varieties and could help to increase the number of parental combinations in citrus cross breeding.

## Conclusion

The present study provides genetic evidence that the CitRKD1 with a RWP-RK domain at the polyembryonic locus plays a principal role in regulating somatic embryogenesis in citrus nucellar tissues. *CitRKD1* alleles were divided into two types, polyembryonic alleles with a MITE insertion and monoembryonic alleles without it. The protein functions of these alleles might be almost identical because of their almost identical amino acid sequences. The transcription of *CitRKD1* might be likely controlled by the MITE insertion in the upstream region, which might act as a transcriptional regulator to alter the innate expression profiles of plant RKD genes with egg cell-specific expression patterns. The information obtained in this study provides a new insight into the apomictic reproduction of heterozygous plants but further analysis is required to clarify whether the MITE insertion can explain how *CitRKD1* acquired broad expression patterns in terms of both time and tissues in contrast to typical plant RKD genes, which have egg cell-specific expression profiles, as well as to address various questions such as what happens in the downstream signalling pathway triggered by *CitRKD1* and how *CitRKD1* have evolved among apomictic plant species.

## Methods

### Plant materials and microarray analysis

‘Kiyomi’ and ‘Harumi’, cultivated at NARO Institute of Fruit and Tea Tree Science (NIFTS), Department of Citrus Research (Shizuoka, Japan), were used as materials for monoembryonic and polyembryonic varieties, respectively. Whole young fruits at 15, 30, 45 and 60 DAF were collected for microarray analysis, immediately frozen in liquid nitrogen, and stored at − 80 °C for RNA isolation. Total RNA was extracted according to a past report [13].

A custom oligo-DNA microarray was designed using the eArray system (Agilent Technologies, Santa Clara, CA, USA; https://earray.chem.agilent.com/earray/) according to the standard system protocols. Probes were constructed using 79 NP-ORFs in the 380 kbp draft sequence of the polyembryonic locus (accession number: AB573149) [28] by the Rice Genome Automated Annotation System (RiceGAAS) (http://ricegaas.dna.affrc.go.jp/) program, and 29,148 assembled mRNAs in the clementine mandarin genome sequence ver. 1.0 (accession number: AMZM001000000) [42]. For the 79 NP-ORFs, probe design was carried out against each exon as far as possible. In total, 29,539 independent probes (391 probes from the polyembryonic locus, 29,148 probes from mRNAs of the clementine mandarin genome sequence) were generated and duplicates were mounted on the custom oligo-DNA microarray (Agilent Design #059979) under the 4 × 44 K format of the Agilent system.

RNA samples were labelled with cyanine-3-labelled cytosine triphosphate using a Low Input Quick-Amp Labelling Kit (Agilent Technologies) according to the manufacturer’s directions. The labelled cRNAs were subsequently hybridized on the custom oligo-DNA microarray using a Gene Expression Hybridization kit (Agilent Technologies) according to the manufacturer’s directions. After washing in GE washing buffer (Agilent Technologies), the glass slides were scanned with an Agilent Microarray Scanner (G2505C, Agilent Technologies). The Feature Extraction software (version 10.5.1.1), employing defaults for all parameters, was used to convert the images into gene expression data. Data analysis was carried out using Subio platform version 1.12 (Subio Inc., Aichi, Japan). The raw data were normalized to the 75th percentile intensity of probes above the background level (gIsWellAbove = 1). The normalized values were filtered based on expression changes under the following conditions: lower signal intensity cut-off < 100, cut-off probes flagged with IsPosAndSignif, expression changes greater than 2-fold differences in the expression ratio between ‘Kiyomi’ and ‘Harumi’ throughout all experiments. GO term enrichment analysis was carried out using the Fisher’s exact test function of the software. The complete microarray data have been deposited in the NCBI Gene Expression Omnibus (https://www.ncbi.nlm.nih.gov/geo/query/acc.cgi?acc=GSE115082) under series entry GSE115082.

### Gene expression analysis of candidate genes in whole young fruits at 15 and 30 DAF of ‘Kiyomi’ and ‘Harumi’ by RT-PCR

cDNA was prepared with 1 μg of purified total RNA using a QuantiTect® Reverse Transcription Kit (Qiagen, Hilden, Germany). The PCR reaction was performed in a ProFlex PCR system (Applied Biosystems, Foster City, CA, USA) thermal cycler using *Ex Taq*® DNA polymerase (Takara, Tokyo, Japan) under the following conditions: 30 cycles of 10 s at 94 °C, 15 s at 56 °C, and 60 s at 72 °C. Primer sequences for four candidate genes were designed using the conserved sequences in the corresponding genes of clementine mandarin and sweet orange in the Phytozome database (https://phytozome.jgi.doe.gov/pz/portal.html). Their primer sequences and those of the control gene *EF1-α* are listed in Additional file 3: Table S3. The PCR products for each reaction were analysed using electrophoresis in 1.5% (*v*/v) agarose gels.

### Isolation and sequencing of cDNA and genomic clones of CitRKD1

Full-length cDNA clones corresponding to NP-ORF44 were obtained from cDNA of satsuma mandarin ovary tissue at anthesis by PCR amplification using primeSTAR® GXL DNA polymerase (Takara). The primer sequences were as follows: forward primer, 5′-TACCTTAAAGAAGCAGCACCACCGA-3′; reverse primer, 5′-GTTTCTTTCAGCTGGTGATCCTT-3′. Genomic DNA was extracted from satsuma mandarin leaves according to a previous report [6]. Genomic clones, including approximately 1.0 kbp of the upstream region and the coding region, were also obtained using the following primer set: forward primer, 5′-AACGAAGGATTAACAGTATTC-3′; reverse primer, 5′-TAATCAGCCTACTTCGTTC-3′. The amplified PCR products were cloned into the pUC118 vector (Takara) using the Mighty cloning reagent set (Takara), and transformed into *Escherichia coli* strain XL-1 Blue. Sanger sequencing analysis was carried out using a BigDye Terminator v. 3.1 Cycle Sequencing Kit (Applied Biosystems) with an ABI PRISM 3100 Genetic Analyzer (Applied Biosystems). Two independent clones corresponding to NP-ORF44 were obtained each from cDNA and genomic DNA of satsuma mandarin, and their complete nucleotide and amino acid sequences were registered in the DNA Data Bank of Japan (DDBJ) (*CitRKD1-mg1*: LC385050, *CitRKD1-mg2*: LC385049)*.* The introns and exons were determined by sequence comparison of genomic and cDNA clones. The deduced amino acid sequences of CitRKD1-mg1 and CitRKD1-mg2 were aligned with other citrus CitRKD1 alleles registered in the public DNA database. Phylogenetic tree analysis was carried out using the neighbour-joining method in the computer program Genetyx-Win Ver. 11.0 (Software Development, Tokyo, Japan), using citrus CitRKD1 alleles, and NLP and RKD genes of clementine mandarin and *Arabidopsis.*

### Expression analysis of CitRKD1 in various tissues of Satsuma mandarin by RT-PCR

Various tissues of satsuma mandarin were sampled including flowers at anthesis, leaves, stems, young whole fruit at 30 DAF and fruit tissues (juice sac and peel) at 60, 120, and 180 DAF. Flowers at anthesis were separated into anthers, pistils, petals, ovaries and sepals. RNA extraction, cDNA preparation and RT-PCR were carried out as described above using the same primer set for NP-ORF44 under the same conditions. The PCR fragment amplified from cDNA of young fruit at 30 DAF was directly sequenced as described above.

### Production of transgenic sweet orange with antisense-overexpression of CuRKD-mg2

The full-length cDNA of *CiRKD1-mg2* was inserted into the *CiFT* co-expression vector [7] in the antisense direction, under the *Cauliflower mosaic virus* (CaMV) 35S promoter. Epicotyl segments of ‘Hamlin’ sweet orange were inoculated with *Agrobacterium tumefaciens* strain LBA4404 according to a previous report [3]. Each adventitious shoot was grafted in vitro onto etiolated Troyer citrange (*C. sinensis* L. × *P. trifoliata* Raf.) seedlings. These grafted plants were re-grafted onto rough lemon (*C. jambhiri* Lush.). Transgene incorporation and Southern blot analyses were carried out according to a previous report [7]. The leaves and flowers of the transgenic sweet orange were sampled and subjected to gene expression analysis for the transgene by RT-PCR under the same conditions for NP-ORF44. The transcription level of endogenous *CitRKD1-org* in the transgenic sweet orange were investigated using the pair of primers as follows: forward primer, 5′-AGAGACTTGATGATTTATTTTCATCTCC-3′ in the fifth exon of *CitRKD1-org*; reverse primer, 5′-TGATCCTTAGCATTAGAGAATGTGT- 3′ in the 3′ untranslated region of *CitRKD1-org*.

The transgenic sweet orange was self-pollinated and mature fruits were harvested at 180 DAF. The seed coats of the transgenic seeds were removed and phenotypic changes of the transgenic seeds were observed. Each seed was separately planted into potting soil and grown in a growth chamber at 25 °C for 4 weeks. Genomic DNA was extracted from the leaves of each germinated T_1_ plant and CAPS analysis was carried out to compare the genotypes of the T_1_ plants between control and transgenic ‘Hamlin’ sweet oranges according to a past report [36]. Five CAPS markers (Cp1624/*Msp*I, Tf0326/*Hha*I, Tf0001/*Msp*I, Mf0097/*Dra*I and Tf0013/*Rsa*I) were selected because the marker loci were in different linkage groups (1, 2, 5, 7 and 8, respectively) and they had heterozygous genotypes in sweet orange. The reaction products were analysed using electrophoresis in 2.0% (*v*/v) agarose gels.

### Association between MITE insertion in the upstream region and transcription of CitRKD1 alleles among 10 representative citrus varieties

Ten representative varieties were investigated to determine whether the MITE insertion affected the transcription of *CitRKD1* as follows; clementine mandarin, ‘Kiyomi’, Mato buntan pumelo, hyuga-natsu and ‘Nishino kaori’ (‘Kiyomi’ × sweet orange) as monoembryonic varieties, and satsuma mandarin, ‘Harumi’, ‘Valencia’ orange, ponkan mandarin and ‘Saga’ mandarin as polyembryonic varieties. The absence or presence of the MITE insertion was evaluated by genomic PCR using the following primer set; forward primer (5′-GTTACTTGGAGACGGCCTAACG-3′) and reverse primer (5′-TCGATCATGTAATGCTGACTC-3′). RT-PCR was carried out under the conditions described above using the same primer set for NP-ORF44.

### Development of a DNA marker discriminating mono/polyembryonic alleles and its application for genotyping of traditional and breeding varieties in Japanese breeding programs

The following 14 ancestral citrus varieties in Japanese breeding programs [14] were used to obtain reference sequences of *CitRKD1* alleles because all alleles in the pedigrees were derived from them; Duncan grapefruit, Dancy tangerine, clementine mandarin, ‘Mukaku-kishu’, Tanigawa buntan pumelo, hassaku, hyuga-natsu, sweet orange, Iyo-kan, satsuma mandarin, ponkan mandarin, Mediterranean mandarin, King mandarin, and ‘Murcott’. Approximately 1.0 kbp to 1.3 kbp fragments including the MITE insertion site in the *CitRKD1* alleles were amplified from their genomic DNA by PCR using primeSTAR® GXL DNA polymerase (Takara), with the same primer set used for genomic PCR described above, and were sequenced. With the aim of precisely evaluating the allelic genotypes of 95 traditional and breeding varieties, a new primer set for a DNA marker (P/M marker) discriminating mono/poly embryonic allelic genotypes was designed with Genetyx-Win ver. 11 (Software Development Co.) using the conserved nucleotide sequences among them. The primer sequence of P/M marker was as follows; sense primer: 5′-TCTCTGGTTCATTGAGAATCC-3′ in the upstream region, antisense primer: 5′-CTGAGCACCAGGCAACAACTAC-3′ in the second exon. Allelic genotyping PCR was carried out using *Ex Taq*® DNA polymerase (Takara) with a program of 30 cycles of 10 s at 98 °C, 5 s at 58 °C, and 30 s at 72 °C in a 25 μl reaction solution. The PCR product for each reaction was analysed using electrophoresis in a 1.5% (*v*/v) agarose gel.

## Additional files

Additional file 1: **Table S1.** Primer sequences of candidate genes on polyembryonic locus for RT-PCR. (XLSX 10 kb)

Additional file 2: **Table S2.** Highly expressed genes in polyembryonic reproductive tissues selected by microarray analysis. (XLSX 24 kb)

Additional file 3: **Table S3.** Weakly expressed genes in polyembryonic reproductive tissues selected by microarray analysis. (XLSX 38 kb)

## Abbreviations

BAC: bacterial artificial chromosome
CAPS: cleaved amplified polymorphic sequences
DAF: days after flowering
MITE: miniature inverted-repeat transposable element
NGS: next-generation sequencing
ORF: open reading frame
RKD: RWP-RKD domain-containing
SNP: single nucleotide polymorphism
SSH: subtractive suppression hybridization
TIR: terminal inserted repeat
TSD: target site duplication
